# Default Mode Network alterations in alexithymia: an EEG power spectra and connectivity study

**DOI:** 10.1038/srep36653

**Published:** 2016-11-15

**Authors:** Claudio Imperatori, Giacomo Della Marca, Riccardo Brunetti, Giuseppe Alessio Carbone, Chiara Massullo, Enrico Maria Valenti, Noemi Amoroso, Giulia Maestoso, Anna Contardi, Benedetto Farina

**Affiliations:** 1 Department of Human Sciences, Università Europea di Roma, Rome, Italy; 2 Sleep Disorders Unit; Institute of Neurology, Catholic University, Rome, Italy

## Abstract

Recent neuroimaging studies have shown that alexithymia is characterized by functional alterations in different brain areas [e.g., posterior cingulate cortex (PCC)], during emotional/social tasks. However, only few data are available about alexithymic cortical networking features during resting state (RS). We have investigated the modifications of electroencephalographic (EEG) power spectra and EEG functional connectivity in the default mode network (DMN) in subjects with alexithymia. Eighteen subjects with alexithymia and eighteen subjects without alexithymia matched for age and gender were enrolled. EEG was recorded during 5 min of RS. EEG analyses were conducted by means of the exact Low Resolution Electric Tomography software (eLORETA). Compared to controls, alexithymic subjects showed a decrease of alpha power in the right PCC. In the connectivity analysis, compared to controls, alexithymic subjects showed a decrease of alpha connectivity between: (i) right anterior cingulate cortex and right PCC, (ii) right frontal lobe and right PCC, and (iii) right parietal lobe and right temporal lobe. Finally, mediation models showed that the association between alexithymia and EEG connectivity values was directed and was not mediated by psychopathology severity. Taken together, our results could reflect the neurophysiological substrate of some core features of alexithymia, such as the impairment in emotional awareness.

The alexithymia construct refers to a personality trait featuring several alterations in emotional processing. It has been theorized as a multidimensional construct associated with (i) difficulty in recognizing and describing one’s emotions, (ii) difficulty in distinguishing between feelings and bodily sensations, (iii) impairment of affect-related fantasy and imagery activity, (iv) externally-oriented thinking (i.e. the predisposition to focus on external stimuli rather than on one’s own internal experiences)^1^. Alexithymia is considered a crucial construct in psychosomatic medicine^2^ and it is related to several psychosomatic sequelae, such as medically unexplained symptoms^3^,^4^, as well as to psychopathology^5^.

From a neurobiological point of view, recent neuroimaging studies have shown that alexithymia is characterized by functional alterations in different brain areas (e.g., the anterior and posterior cingulate cortex), during emotional/social tasks as well as in imagery and somatosensory tasks^6^. On the other hand, electroencephalographic (EEG) studies in alexithymic subjects during emotional tasks (e.g. EEG connectivity and event-related potential studies) documented several abnormalities in the functional integration between brain areas (i.e. between anterior and posterior cortical regions), predominantly in the right hemisphere^7^,^8^,^9^,^10^,^11^.

As concerns cortical networking features of alexithymic subjects during resting state (RS), few data are available. RS network activity can be defined as *“coherent and spontaneous fluctuations of human brain activity in distinct and spatially separate networks of varying granularity when subjects are not engaged in a particular task or superior cognitive processes*”^12^. The default mode network (DMN) is most frequently detected during the RS condition^12^ and reflects the neural activity of different brain areas such as cingulate cortex, hippocampus, medial frontal lobes, inferior parietal lobes, and temporal lobes^13^,^14^. It is thought to be involved in self-consciousness, self-processing and introspection functions, including emotional awareness and processing^15^, which are supposed to be impaired in alexithymia^16^. Recently, a consistent link between alterations in emotional processing and reduced DMN connectivity has been detected^16^,^17^. Furthermore, abnormalities in DMN activity and its functional connectivity have been widely reported in several psychiatric disorders, such as schizophrenia and mood disorders^18^.

Although functional magnetic resonance imaging (fMRI) is commonly used to investigate the functional connectivity of DMN, recent studies have shown that also EEG is suitable to investigate this network^12^,^14^ *“assessing changes in the synaptic synchrony of millions of neurons connected at varying time delays and frequencies*”^14^. Furthermore, compared to fMRI, EEG time-series data directly relate to dynamic postsynaptic activity in the cerebral cortex with a higher temporal resolution^19^. Conversely, MR-based methods cannot asses fast-frequency synchronized neuronal activity^20^. Finally, the EEG offers a valuable tool to assess *“real-time electrical activity in the brain and is overall a less costly, time-consuming, and complex procedure*”^21^.

To the best of our knowledge, only one study has directly investigated the DMN in subjects with alexithymia. Liemburg *et al*.^16^, in an fMRI study, showed that, compared to non-alexithymic participants, alexithymic individuals showed lower connectivity in medial frontal and temporal areas. Therefore, the main aim of the present study was to extend these previous findings by exploring the modifications of EEG power spectra and EEG functional connectivity in DMN in subjects with alexithymia. Furthermore, another aim was to investigate the association among any significantly modified connections of DMN, alexithymia severity and general psychopathology.

## Materials and Methods

### Participants

Participants were recruited from the Università Europea di Roma through advertisements posted at the university. The enrollment lasted from October 2015 to March 2016. Ninety-five subjects who agreed to participate were administered the 20-item Toronto Alexithymia Scale (TAS-20)^22^,^23^, the revised version of Symptom Check list 90 (SCL-90-R)^24^ and a checklist assessing socio-demographic data and the study inclusion criteria. Inclusion criteria were: right handedness; no history of medical, psychiatric and neurologic diseases; head trauma; no assumption of Central Nervous System active drugs in the two weeks prior to study entry.

After receiving information about the aims of the study, all participants provided a written consent to participate in the study that was performed according to the Helsinki declaration standards and was approved by the Università Europea’s ethics review board. Informed consent has been obtained for all participants involved in the present study.

Eighteen consecutive subjects (three men and fifteen women, mean age: 25.00 ± 8.12 years) with TAS-20 total score ≥58 were enrolled in the alexithymia group (TAS+ group). According to a case-control design, we enrolled a control group consisting of eighteen subjects matched for sex and age (three men and fifteen women, mean age: 23.89 ± 4.85) with low alexithymia (TAS-20 total score ≤45, TAS− group). The cut-off values of TAS-20 used for selection of study groups were those adopted by Romei and coworkers^25^. Clinical and socio-demographic characteristics of the final sample are listed in Table 1.

### Questionnaires

The TAS-20^22^,^23^ is a 20-item self-report that is one of the most commonly used measures of alexithymia. Items are rated on a 5-point Likert scale (from 1 = Strongly disagree to 5 = Strongly agree) indicating the degree to which each statement describes the respondent’s behavior. It contains the following three subscales, theoretically formulated and confirmed through factor analysis^23^: (1) Difficulty Describing Feelings (DDF) refers to difficulty describing and communicating emotions, (2) Difficulty Identifying Feeling (DIF) refers to difficulty identifying emotions, (3) Externally-Oriented Thinking (EOT) concerns the tendency of individuals to focus their attention externally. The TAS total score (ranging from 20 to 100) corresponds to the general level of alexithymia. In the present study we used the Italian version of the TAS^26^. The Cronbach’s α in the present sample was 0.90 for the TAS-20 total score.

The SCL-90-R^24^ is a 90-item questionnaire on 5-point Likert scale (0–4) assessing nine primary symptom dimensions: somatization (SOM), obsessive-compulsive symptoms (O-C), interpersonal sensitivity (I-S), depression (DEP), anxiety (ANX), hostility (HOS), phobic anxiety (PAR), paranoid ideation (PAR) and psychoticism (PSY). Furthermore, seven additional items assess disturbances in appetite and sleep (OTHER). The SCL-90-R also provides a global severity index (GSI) which is designed to measure overall psychological distress. Higher scores indicate more psychological symptoms in each subscale as well as a higher degree of distress, higher intensity of symptoms, and more self-reported symptoms^27^. In the present study, a previously validated Italian version of the scale was used^27^ and the Cronbach’s alpha in the present sample was 0.92.

### EEG recordings

RS recordings were performed in an EEG Laboratory, with each subject sitting in a comfortable armchair, with his/her eyes closed, in a quiet, semi-darkened silent room for 5 minutes. In order to avoid alcohol and or caffeine effects on EEG data^28^,^29^, participants were asked to refrain from drinking alcohol and caffeine for 4 to 6 hours immediately before their EEG recordings.

EEG was recorded by means of a Micromed System Plus digital EEGraph (Micromed^©^ S.p.A., Mogliano Veneto, TV, Italy). EEG montage included 19 standard scalp leads positioned according to the 10–20 system (recording sites: Fp1, Fp2, F7, F3, Fz, F4, F8, T3, C3, Cz, C4, T4, T5, P3, Pz, P4, T6, O1, O2), Electrooculography (EOG) and Electrocardiography (EKG). The reference electrodes were placed on the linked mastoids. Impedances were kept below 5 KΩ before starting the recording and checked again at the end of the experimental recording. Sampling frequency was 256 Hz; A/D conversion was made at 16 bit; pre-amplifiers amplitude range was ±3200 μV and low-frequency pre-filters were set at 0.15 Hz. The following band-pass filters were used: high frequency filters (HFF) = 0.2 Hz; low frequency filters (LFF) = 128 Hz.

Artifact rejection (eye movements, blinks, muscular activations, or movement artifacts) was performed visually on the raw EEG. The recordings were attended by trained technicians, and the simultaneous recording of EOG and EKG further improved the artifact recognition and removal (more details of artifact rejection procedure could be found in refs 30,31). At least 120 seconds of EEG artifact-free recording (not necessarily consecutive) were analyzed for each group, in all conditions. The average time analyzed was 276 ± 18 sec., 280 ± 21 sec. respectively for TAS+ and TAS− subjects. All EEG analysis were performed by means of the exact Low Resolution Electric Tomography software (eLORETA) software, a validated tool for localizing the electric activity in the brain based on multichannel surface EEG recordings^32^. The eLORETA software benefits from an excellent localization agreement with different multi-modal imaging techniques, also when standard 19-electrodes EEG montage was used^14^.

### Frequency analysis

Fast Fourier Transform algorithm was used to perform EEG frequency analysis, with 2 seconds interval on the EEG signal, in all scalp locations. In the present study we have considered the following frequency bands: delta (0.5–4 Hz); theta (4.5–7.5 Hz); alpha (8–12.5 Hz); beta (13–30 Hz); and gamma (30.5–60 Hz). EEG frequency analysis was performed using monopolar EEG traces (each electrode referred to joint mastoids). Topographic sources of EEG activities were determined using the eLORETA software. This software calculates the 3-dimensional current distribution throughout the brain volume by assuming that neighboring neurons are activated both simultaneously and synchronously. This is in accordance with results of single cell recordings in the brain^33^,^34^. The computational task is to select the smoothest 3-dimensional current distribution, which is a widely used procedure in signal processing^35^,^36^. The result is a true 3-dimensional tomography, in which the localization of brain signals is conserved with a low amount of dispersion^37^.

### Connectivity analysis

The connectivity analysis was performed by the computation of lagged phase synchronization. This connectivity measure is widely used to assess EEG functional connectivity in both psychiatric and brain diseases^38^,^39^. Furthermore, compared to other connectivity measure, the lagged phase synchronization has some advantages (e.g. is resistant to non-physiological artifacts and it is minimally affected by low spatial resolution)^37^,^40^.

The eLORETA software computes lagged phase synchronization *ρ*_x,y_(*ω*), by the formula^41^:


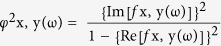


Details on eLORETA lagged phase synchronization formula can be found in Pascual-Marqui’s studies^37^,^41^.

In order to evaluate the connectivity in the DMN, 12 Regions of Interest (ROIs) were defined according to Thatcher and coworkers^14^ (Table 2). These Authors selected the Brodmann’s areas involved in the DMN on the basis of the review by Buckner *et al*.^13^. The ‘single nearest voxel’ option was chosen because eLORETA has low spatial resolution, and the single centroid voxel is considered an excellent representative of the ROI^37^. In this way, each ROI consisted of a single voxel, the closest to each seed.

The eLORETA computed the lagged phase synchronization values between all these ROIs (total 12 × 12 = 144 connections). The eLORETA also computed the source reconstruction algorithm previously described^32^,^42^. The EEG connectivity analysis was performed on the same blocks of EEG tracings used for power spectra analysis.

### Statistical analysis

The power spectra analysis and EEG connectivity were compared between TAS+ and TAS− participants for each frequency band. The comparisons were performed using the statistical non-parametric mapping (SnPM) methodology supplied by the eLORETA software^43^. This methodology is based on the Fisher’s permutation test (i.e., a subset of non-parametric statistics, more details could be found in ref. 43). Correction of significance for multiple testing was computed for the comparisons between TAS+ and TAS− groups for each frequency band. The non-parametric randomization procedure, available in the eLORETA program package, was performed for the correction of multiple comparison (i.e., Holmes’ non-parametric correction for multiple comparisons^43^,^44^). The T-level thresholds were computed by the statistical software implemented in the eLORETA, which correspond to statistically significant thresholds (*p* < 0.05 and *p* < 0.01)^45^. These T-level thresholds and the correspondent *p* values were provided after applying the correction for multiple comparisons.

Two-way chi-squared and Kolmogorov-Smirnov Z test were used to analyze differences between groups, respectively, for N × N contingency tables and dimensional measures. Spearman’s *rho* correlation coefficients were reported as measures of associations among the TAS total scores, TAS sub-scales, SCL-90, along with any significantly modified connections between ROIs. Due to the strong relationship between alexithymia and psychopathology^5^, the GSI was included as a control variable in a partial correlation (*r*_p_) analyses. Furthermore, to determine whether the relationship between alexithymia and EEG connectivity data was mediated by psychopathology, multiple mediation analysis was performed using the “process” macro for SPSS^46^. This tool assesses direct and indirect effects of one or more independent variables (IV) on a dependent variable (DV) through one or more mediators (M). In the present study we tested a model in which alexithymia severity (TAS total score) is the independent variable, EEG connectivity values (interconnected ROIs) are the dependent variables, and psychopathology (GSI) is a potential mediator. It is important to note that the design of this analysis is correlational in nature, which precludes a causal interpretation of the association between these variables. In the present analyses, we used standardized variables to generate standardized coefficients and corresponding *p* values. As suggested by Preacher and Hayes^47^, for indirect effects we also calculated bias-corrected and accelerated 95% CI produced using a bootstrapping method.

Two-way chi-squared test, Kolmogorov-Smirnov Z test and correlation analyses were performed using IBM SPSS Statistics for Windows, version 19.0.

## Results

EEG recordings suitable were obtained for all participants. Qualitative visual evaluation of the EEG recordings showed no relevant modifications of the background rhythm frequency (e.g. focal abnormalities or epileptic discharges) and no evidence of drowsiness or sleep during the recordings. Differences between groups (TAS+ subjects vs. TAS− subjects) are reported in Table 1. Compared to TAS− subjects, TAS+ individuals reported higher mean scores on the GSI and on six (SOM, O-C, DEP, AXN, HOST, PSY) of the ten dimensions of the SCL-90-R. No significant differences were found for age and for substance use (alcohol and tobacco in the last 6 months) between the two groups.

### Power spectra analysis

**Figure 1:**
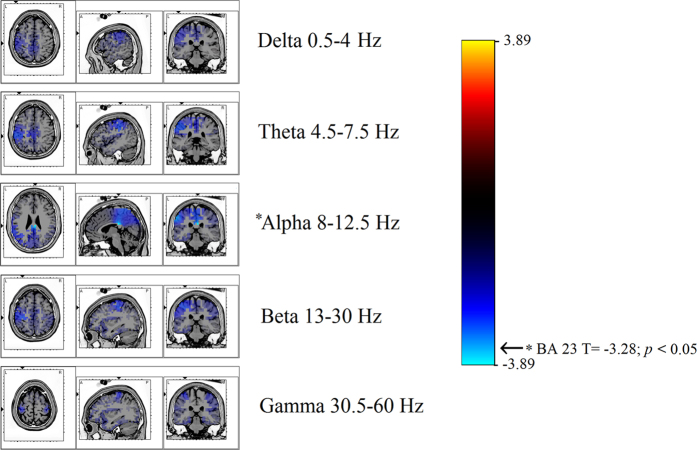
Results of the eLORETA comparison of EEG power spectra in each frequency band. Threshold values (T) for statistical significance (corresponding to *p* < 0.05) are reported in the right side of the figure. Blue color indicates reduction of EEG power spectra. Red color (not present) would indicate an increase of EEG power spectra. Compared to TAS− individuals, TAS+ subjects showed a decrease of alpha power in the in right PCC (BA 23; T = −3.28, *p* < 0.05). Abbreviation: BA = Brodmann areas; PCC = Posterior Cingulate Cortex; A = Anterior; P = Posterior; L = Left; R = Right.

In this analysis the thresholds for significance were T = ±3.23 corresponding to *p*  < 0.05, and T = ±3.89, corresponding to *p* < 0.01. A significant modification was observed in the alpha band: compared to TAS− individuals, TAS+ subjects showed a decrease of alpha power in the limbic lobe (Fig. 1). The eLORETA software localized these modifications in right PCC (Brodmann Area, BA 23; T = −3.28, *p* < 0.05). No significant differences were observed in the other frequency bands.

### Connectivity analysis

**Figure 2:**
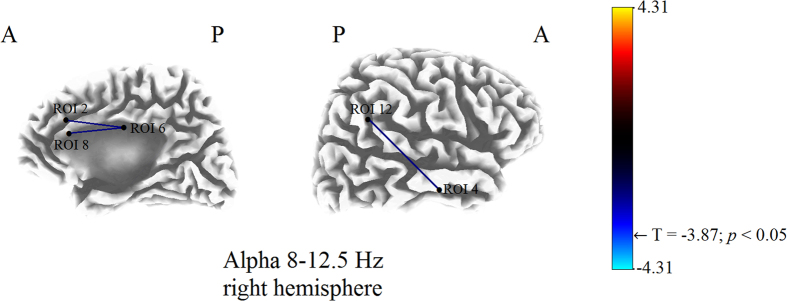
Results of the eLORETA comparison of EEG lagged phase synchronization in each frequency bands. Threshold values (T) for statistical significance (corresponding to *p* < 0.05) are reported in the right side of the figure. Blue lines indicate connections which presented decrease of connectivity. Red lines (not present) would indicate an increase of connectivity. In this analysis, compared to TAS− individuals, TAS+ subjects showed a decrease of alpha lagged phase synchronization between: (i) ROI 6 (BAs 23–24) and ROI 8 (BA 32) (T = −3.87; *p* < 0.05), (ii) ROI 2 (BAs 8-9-10) and ROI 6 (BAs 23–24) (T = −4.10; *p* < 0.05), and (iii) ROI 12 (BAs 39–40) and ROI 4 (BAs 21-28-36) (T = −3.92; *p* < 0.05). Abbreviation: A = Anterior; P = Posterior; ROI = Region of interest.

In this analysis the thresholds for significance were T = ±3.72 corresponding to *p* < 0.05, and T = ±4.31, corresponding to *p* < 0.01. Significant modifications were observed in the alpha band (Fig. 2). Compared to TAS− individuals, TAS+ subjects showed a decrease of alpha lagged phase synchronization between: (i) ROI 6 (BAs 23–24) and ROI 8 (BA 32) (T = −3.87; *p* < 0.05), (ii) ROI 2 (BAs 8-9-10) and ROI 6 (BAs 23–24) (T = −4.10; *p* < 0.05), and (iii) ROI 12 (BAs 39–40) and ROI 4 (BAs 21-28-36) (T = −3.92; *p* < 0.05).

### Association among modified connections between Regions of Interest (ROIs), TAS and SCL-90-R

TAS total scores were negatively associated with the modified connections between ROIs 2–6 (*rho* = −0.50; *p* < 0.01), ROIs 8–6 (*rho* = −0.52; *p* < 0.01), and ROIs 12-4 (*rho* = −0.39; p < 0.05). Furthermore, significant correlations were also observed between TAS subscales and interconnected ROIs. The association among TAS total scores, and modified connections between ROIs 2–6 (*r*_p_ = −0.39; *p* < 0.05), ROIs 8–6 (*r*_p_ = −0.44; *p* < 0.01), and ROIs 12-4 (*r*_p_ = −0.39; *p* < 0.05) were significant after controlling for GSI. Correlations and partial correlations are respectively reported in Tables 3 and 4.

**Figure 3:**
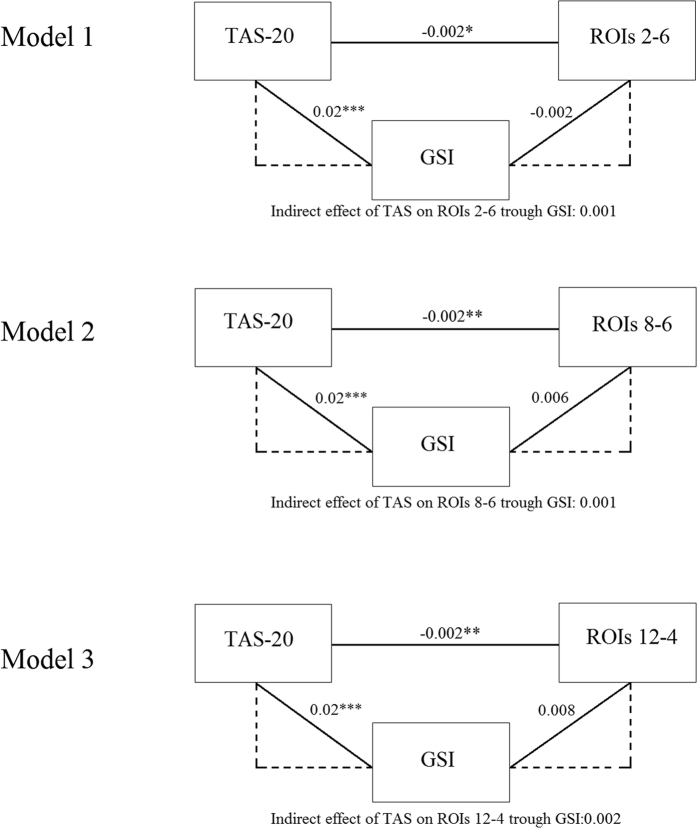
Results of the multiple mediation models and related standardized *b* coefficients. Note. *p < 0.05; **p < 0.01; ***p < 0.001. Abbreviation: TAS-20 = Toronto Alexithymia Scale; GSI = Global Severity Index; ROIs = Regions of interest.

The mediation models explained between 15% (F_2;33_ = 3.96; *p* < 0.05; Fig. 3 Model 3) and 20% (F_2;33_ = 5.31; *p* < 0.01; Fig. 3 Model 2) of the data variability. All mediation models indicated that the total effect of TAS-20 on EEG functional connectivity values was significant (b = −0.01; *p* < 0.01), with more severe alexithymia associated with a greater decrease in EEG functional connectivity value. Moreover, the relationship between alexithymia and EEG functional connectivity data was directed and was not mediated by psychopathology severity [*b* between 0.001 (*p* = 0.92) and 0.002 (*p* = 0.69)]. Finally, in all mediational models no significant direct effect was observed for GSI on EEG functional connectivity values (Fig. 3). Detailed statistics of the mediational models are listed in Table 5.

## Discussion

The main aim of the present study was to explore the modifications of EEG power spectra and EEG functional connectivity in DMN in alexithymia. Compared to TAS− individuals, TAS+ subjects showed a decrease of alpha power in the right PCC (BA 23). Furthermore, compared to TAS− individuals, TAS+ subjects showed a decrease of alpha connectivity between: (i) right ACC and right PCC, (ii) right frontal lobe and right PCC, and (iii) right parietal lobe and right temporal lobe. EEG connectivity values were also negatively related to TAS total score after controlling for general psychopathology, which is known to be associated with alexithymia^5^. Finally, our mediation models results showed that the relationship between alexithymia and EEG functional connectivity value was directed (i.e., more severe alexithymia associated with a greater decrease in EEG functional connectivity) and was not mediated by psychopathology severity.

Taken together, our results could reflect the neurophysiological substrate of several core features of alexithymia, such as the impairment in emotional awareness as well as the alexithymia-related imagery deficit. Our results are consistent with previous EEG and fMRI studies suggesting that alexithymia is characterized by a disruption in the integrated cortical neural network not only during emotional tasks^7^,^10^,^11^ but also during RS condition^16^. Our study differs from and adds to previous findings by investigating EEG modifications during RS condition, which is thought to reflect intrinsic activity in the brain revealing valuable information on how different structures communicate^48^.

Our results are in line with previous studies reporting the involvement of alpha frequency in alexithymia. Indeed, the decrease of both EEG power and EEG connectivity in the alpha frequency band was previously detected in alexithymic subjects in response to emotional visual stimuli^9^,^10^. Due to the strong association between alpha frequency and cognitive processing related to external attention^49^,^50^, it has been hypothesized that this neurophysiological pattern could reflect the externally-oriented thinking of alexithymic subjects^9^.

Our data are also in accordance with previous functional imaging studies confirming the crucial role of the cingulate cortex in alexithymia (for a review see refs 6,51). The PCC has shown to be involved in several emotional and cognitive processing^52^. It is also considered the crucial node of the DMN^53^, with critical relevance in maintaining a sense of self-consciousness. Moreover, PCC is engaged in self-referential thoughts during RS^54^. In a fMRI study, Mantani *et al*.^55^ reported a significant decrease of PCC activation in subjects with high degrees of alexithymia during imagery tasks, suggesting a possible involvement of PCC in alexithymia-related imagery disturbance. On the other hand, the involvement of ACC in alexithymia is also well documented^6^,^51^. A reduction of ACC activity in response to emotional stimuli (especially for emotionally negative stimuli) was observed in several neuroimaging studies^56^,^57^,^58^. Alterations in the cingulate cortex in subjects with alexithymia ware also observed by Liemburg *et al*.^16^ during RS condition. Therefore, the decrease of functional connectivity between ACC and PCC observed in our study could reflect both affective and cognitive disturbances in alexithymia (i.e. the impairment in emotional awareness and the externally-oriented thinking). This hypothesis is also in accordance with our correlational data (i.e., negative correlation between ACC/PCC connectivity and both DDF and EOT TAS-20 factors). Compared to Liemburg *et al*.^16^, we did not observe an increase of functional connectivity in brain areas involved in sensory input. The authors, using Independent Component Analysis (i.e., a data-driven method that separate the fMRI signal into spatially independent networks), showed that within the DMN, compared to controls, alexithymic participant showed higher functional connectivity in the precentral gyrus and occipital areas suggesting a tendency of the alexithymic towards strong bodily expressions of emotions^16^. Moreover, Liemburg *et al*. also did not detect a lateralization. The discrepancies between these results and the present research may be explained by differences in their study designs and methods (i.e., EEG vs fMRI, ROIs selections etc.).

In TAS+ subjects, a decrease of alpha connectivity was also observed between right frontal lobe and right PCC. The role of frontal areas, especially of the prefrontal cortex (PFC), in alexithymia is still unclear^51^. Although PFC plays a crucial role in several cognitive and emotional functions, such as decision-making and emotion regulation^59^, only few studies reported the association between PFC activity and alexithymia. In a Positron Emission Tomography (PET) study of Kano *et al*.^56^, the alexithymic, compared to non-alexithymic subjects, showed decreased activation of right frontal areas (i.e. inferior and superior frontal cortex and the orbital PFC), in response to negative emotional stimuli. Consistently, lower connectivity within the DMN for the superior frontal gyri in alexithymic individuals was also observed^16^. The PCC connections with PFC are thought to be crucial in the DMN^13^ and, as already mentioned, the decrease of connectivity between prefrontal regions and cingulate gyrus could reflect the emotional awareness disturbances, especially “*the difficulties to put feelings into words*”^16^.

We also observed a reduction of EEG connectivity between the right inferior parietal lobule (IPL) and the right temporal lobe. It has been observed that increased connectivity in these areas is associated with envisioning future tasks (i.e., imagine a future situation related to a particular cue, for a review see ref. 13). Therefore, a connectivity decrease between IPL and right temporal lobe may reflect the impairment of affect-related fantasy and imagery activity frequently observed in alexithymic individuals.

Finally, we detected a decrease of EEG alpha power and EEG connectivity in the right hemisphere. It has been hypothesized that alexithymia is associated with a disturbance in right hemisphere functioning, especially in the processing of emotions^60^,^61^. Therefore, our results seem to be in line with both behavioral studies^62^ and neuroimaging data^51^,^63^ suggesting a right hemisphere alteration in alexithymic individuals. The decrease of EEG connectivity and EEG power in the right hemisphere observed in alexithymic subjects is also in accordance with Bucci’s model^64^, suggesting that this neurophysiological pattern may reflect the impairment of the symbolization of emotions, as well as the disturbances in holistic and nonverbal processing of incoming stimuli. It is important to note that our interpretations remain speculative due to the absence of imaginative/emotional tasks in the present study. However, it has been observed that RS activity may predict modifications in behavioral performance and task-evoked brain activity^65^.

There are some limitations in generalizing our results. One limitation that must be considered is that the sample we used is small, and this is especially relevant concerning mediation analysis. Our sample also included mostly female participants. Although conclusive EEG data on DMN gender differences are not yet available, previous studies suggested that women may show different EEG RS brain activity^66^. Therefore, we cannot exclude that women with alexithymia, compared to men with alexithymia, may be characterized by different EEG power and connectivity patterns during RS. Furthermore, we used scalp EEG recordings, which have an intrinsic limit in space resolution. It should be also noted that, although the SCL-90-R is considered a good tool to assess general psychopathology, it does not exclude the presence of a psychiatric disorder. Finally, we have investigated the DMN in a non-clinical sample of alexithymic subjects. Therefore, it is possible that psychiatric patients with alexithymia have different EEG patterns. Although these ideas are purely hypothetical, they might be useful in guiding future research.

Despite these limitations, to the best of our knowledge, this is the first study which simultaneously investigated EEG functional connectivity and EEG power spectra during RS condition in alexithymic subjects, controlling for psychopathology and using an accurate and validated tool (i.e., eLORETA) to localize electric activity in the brain. In conclusion, our results suggest that alexithymia is characterized by a disruption of cortical neural networks, not only during emotional processing, but also during RS condition. This is in accordance with previous hypotheses suggesting that *“such distinct patterns of connectivity may be related to the diminished emotional awareness of alexithymic people*”^16^. Therefore, our results could be of some clinical relevance both for diagnosis and therapy. On one hand, if confirmed, this EEG pattern could help to measure alexithymia in RS condition; on the other hand it highlights the possibility of developing new therapeutic approaches focused on the self neuro-modulation, such as alpha training neurofeedback.

## Additional Information

**How to cite this article**: Imperatori, C. *et al*. Default Mode Network alterations in alexithymia: an EEG power spectra and connectivity study. *Sci. Rep.* **6**, 36653; doi: 10.1038/srep36653 (2016).

**Publisher’s note**: Springer Nature remains neutral with regard to jurisdictional claims in published maps and institutional affiliations.

## Figures and Tables

**Table 1:** Demographic and clinical data of participants.

	TAS+ (n = 18)	TAS− (n = 18)	test	*p*
Variables				
Age - *M (SD*)	25.00 ± 8.12	23.89 ± 4.85	Z-test = 0.33	0.90
Alcohol use in the last 6 months - N (%)	8 (44.4%)	11 (61.1%)	χ^2^_1_ = 1.01	0.32
Tobacco use in the last 6 months - N (%)	7 (38.6%)	8 (44.4%)	χ^2^_1_ = 1.11	0.74
TAS-20				
TAS Total Scores - *M (SD*)	61.11 ± 5.11	36.83 ± 7.02	Z-test = 3	<0.001
DDF subscale - *M (SD*)	19.33 ± 2.01	9.50 ± 3.13	Z-test = 3	<0.001
DIF subscale - *M (SD*)	22.17 ± 4.06	12.11 ± 3.69	Z-test = 2.5	<0.001
EOT subscale - *M (SD*)	19.61 ± 4.35	15.22 ± 2.46	Z-test = 1.83	<0.001
SCL-90-R				
GSI - *M (SD*)	0.93 ± 0.45	0.47 ± 0.38	Z-test = 1.5	<0.05
SOM - *M (SD*)	1.07 ± 0.62	0.51 ± 0.46	Z-test = 1.5	<0.05
O-C - *M (SD*)	1.29 ± 0.65	0.58 ± 0.48	Z-test = 1.68	<0.01
I-S - *M (SD*)	0.88 ± 0.45	0.49 ± 0.42	Z-test = 1	0.27
DEP - *M (SD*)	1.10 ± 0.69	0.60 ± 0.54	Z-test = 1.5	<0.05
ANX - *M (SD*)	1.08 ± 0.64	0.56 ± 0.43	Z-test = 1.5	<0.05
HOS - *M (SD*)	0.96 ± 0.84	0.35 ± 0.36	Z-test = 1.5	<0.05
PHOB - *M (SD*)	0.37 ± 0.53	0.21 ± 0.37	Z-test = 0.68	0.77
PAR - *M (SD*)	0.92 ± 0.55	0.42 ± 0.50	Z-test = 1.68	<0.01
PSY - *M (SD*)	0.50 ± 0.41	0.21 ± 0.35	Z-test = 1.5	<0.05
OTHER - *M (SD*)	1.04 ± 0.82	0.90 ± 1.03	Z-test = 0.68	0.77

Note: TAS-20 = Toronto Alexithymia Scale; DDF = Difficulty Describing Feelings; DIF = Difficulty Identifying Feeling; EOT = Externally-Oriented Thinking; SCL-90-R = Symptom Check list-90-Revised; GSI = Global Severity Index; SOM = somatization; O-C = obsessive-compulsive symptoms; I-S = interpersonal sensitivity; DEP = depression; ANX = anxiety; HOS = hostility; PHOB = phobic anxiety; PAR = paranoid ideation; PSY = psychoticism.

**Table 2:** Cortical 12 regions of interest (ROIs).

ROI	eLORETA MNI coordinates			Anatomical regions	Brodmann areas
	*x*	*y*	*z*		
1	−30	40	25	Left Frontal Lobe	8-9-10
2	20	35	30	Right Frontal Lobe	8-9-10
3	−45	−15	−25	Left Temporal Lobe	21-28-36
4	55	−15	−20	Right Temporal Lobe	21-28-36
5	−5	−5	35	Left Posterior Cingulate Cortex	23–24
6	5	−10	30	Right Posterior Cingulate Cortex	23–24
7	−5	30	20	Left Anterior Cingulate Cortex	32
8	5	30	20	Right Anterior Cingulate Cortex	32
9	−5	−55	25	Left Hippocampus	29-30-31
10	5	−50	25	Right Hippocampus	29-30-31
11	−45	−50	40	Left Parietal Lobe	39–40
12	45	−50	35	Right Parietal Lobe	39–40

Adapted from Thatcher *et al*.^14^.

Note: ROI = Region of Interest; eLORETA = exact Low Resolution Electric Tomography software; MNI = Montreal Neurological Institute.

**Table 3:** Values of Spearman’s *rho* correlation coefficient among values of interconnected lagged phase synchronization ROIs, TAS-20 and GSI scores in all sample (N = 36).

	TAS-20 Total scores	DDF	DIF	EOT	GSI	ROIs 2–6	ROIs 8–6	ROIs 12–4
**TAS-20 Total score**	—							
**DDF**	0.89**	—						
**DIF**	0.90**	0.85**	—					
**EOT**	0.57**	0.44**	0.33*	—				
**GSI**	0.60**	0.61**	0.64**	0.24	—			
**ROIs 2–6**	−0.50**	−0.42**	−0.49**	−0.51**	−0.37*	—		
**ROIs 8–6**	−0.52**	−0.45**	−0.50**	−0.46**	−0.31	0.93**	—	
**ROIs 12-4**	−0.39*	−0.43*	−0.44**	−0.33*	−0.25	0.66**	0.64**	—

Significant correlations are indicated by stars (*).

Note: *p < 0.05; **p < 0.01; TAS-20 = Toronto Alexithymia Scale; DDF = Difficulty Describing Feelings; DIF = Difficulty Identifying Feeling; EOT = Externally-Oriented Thinking; GSI = Global Severity Index.

**Table 4:** Values of partial correlation’s coefficient (controlled for GSI) among values of interconnected lagged phase synchronization ROIs and TAS-20 in all sample (N = 36).

Controlling for		ROIs 2–6	ROIs 8–6	ROIs 12–4
**GSI**	**TAS-20 total score**	−0.39*	−0.44**	−0.39*
	**DDF**	−0.32*	−0.35*	−0.37*
	**DIF**	−0.26	−0.33*	−0.35*
	**EOT**	−0.37*	−0.39*	−0.23

Significant correlations are indicated by stars (*).

Note: *p < 0.05; **p < 0.01; TAS-20 = Toronto Alexithymia Scale; DDF = Difficulty Describing Feelings; DIF = Difficulty Identifying Feeling; EOT = Externally-Oriented Thinking; GSI = Global Severity Index.

**Table 5:**
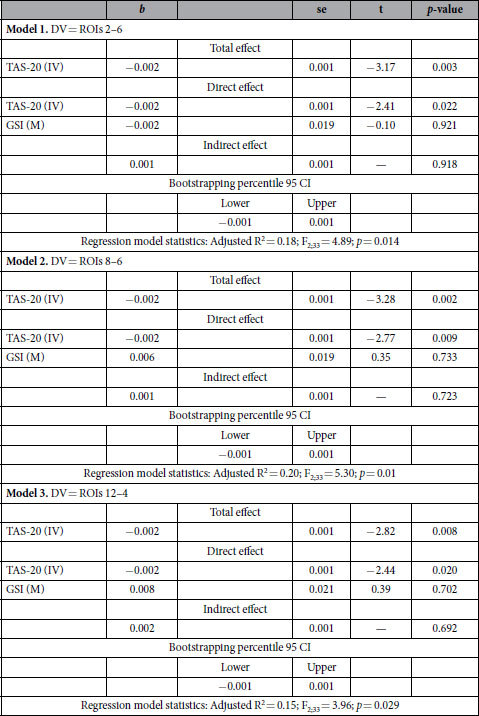
Statistical results of mediation models.

Abbreviation: IV = Independent variable; DV = Dependent variable; M = Mediator; TAS-20 = Toronto Alexithymia Scale; GSI = Global Severity Index; ROIs = Regions of interest; CI = Confidence interval.

## References

[b1] Taylor G. J. Recent developments in alexithymia theory and research. Can J Psychiatry 45, 134–142 (2000). 10742872 10.1177/070674370004500203

[b2] Komaki G. In Somatization and Psychosomatic Symptoms (ed Koh K. B.) 41–50 (Springer, 2013).

[b3] De Gucht V. Neuroticism, alexithymia, negative affect and positive affect as predictors of medically unexplained symptoms in primary care. Acta Neuropsychiatr 14, 181–185, doi: 10.1034/j.1601-5215.2002.140404 (2002). 26984330

[b4] De Gucht V., Fischler B. & Heiser W. Neuroticism, alexithymia, negative affect, and positive affect as determinants of medically unexplained symptoms. Personality and Individual Differences 36, 1655–1667 (2004).

[b5] Grabe H. J., Spitzer C. & Freyberger H. J. Alexithymia and personality in relation to dimensions of psychopathology. Am J Psychiatry 161, 1299–1301, doi: 10.1176/appi.ajp.161.7.1299 (2004). 15229067

[b6] Moriguchi Y. & Komaki G. Neuroimaging studies of alexithymia: physical, affective, and social perspectives. Biopsychosoc Med 7, 8, doi: 10.1186/1751-0759-7-8 (2013). 23537323 PMC3621096

[b7] Aftanas L. I., Varlamov A. A., Reva N. V. & Pavlov S. V. Disruption of early event-related theta synchronization of human EEG in alexithymics viewing affective pictures. Neurosci Lett 340, 57–60, doi: S0304394003000703 (2003). 12648758 10.1016/s0304-3940(03)00070-3

[b8] Aftanas L. & Varlamov A. Associations of alexithymia with anterior and posterior activation asymmetries during evoked emotions: EEG evidence of right hemisphere “electrocortical effort”. Int J Neurosci 114, 1443–1462 (2004). 15636355 10.1080/00207450490509230

[b9] Aftanas L. I. & Varlamov A. A. Effects of alexithymia on the activity of the anterior and posterior areas of the cortex of the right hemisphere in positive and negative emotional activation. Neurosci Behav Physiol 37, 67–73, doi: 10.1007/s11055-007-0151 (2007). 17180321

[b10] Houtveen J. H., Elton M. R. & Bermond B. Alexithymia: A disruption in a cortical network? An EEG power and coherence analysis. Journal of Psychophysiology 11, 147–157 (1997).

[b11] Matsumoto A., Ichikawa Y., Kanayama N., Ohira H. & Iidaka T. Gamma band activity and its synchronization reflect the dysfunctional emotional processing in alexithymic persons. Psychophysiology 43, 533–540, doi: 10.1111/j.1469-8986.2006.00461 (2006). 17076809

[b12] Neuner I. . The default mode network and EEG regional spectral power: a simultaneous fMRI-EEG study. PLoS One 9, e88214, doi: 10.1371/journal.pone.0088214 (2014). 24505434 PMC3914938

[b13] Buckner R. L., Andrews-Hanna J. R. & Schacter D. L. The brain’s default network: anatomy, function, and relevance to disease. Ann N Y Acad Sci 1124, 1–38, doi: 10.1196/annals.1440.011 (2008). 18400922

[b14] Thatcher R. W., North D. M. & Biver C. J. LORETA EEG phase reset of the default mode network. Front Hum Neurosci 8, 529, doi: 10.3389/fnhum.2014.00529 (2014). 25100976 PMC4108033

[b15] Schilbach L., Eickhoff S. B., Rotarska-Jagiela A., Fink G. R. & Vogeley K. Minds at rest? Social cognition as the default mode of cognizing and its putative relationship to the “default system” of the brain. Conscious Cogn 17, 457–467, doi: 10.1016/j.concog.2008.03.013 (2008). 18434197

[b16] Liemburg E. J. . Altered resting state connectivity of the default mode network in alexithymia. Soc Cogn Affect Neurosci 7, 660–666, doi: 10.1093/scan/nss048 (2012). 22563009 PMC3427871

[b17] Shi H. . Default mode network alterations during implicit emotional faces processing in first-episode, treatment-naive major depression patients. Front Psychol 6, 1198, doi: 10.3389/fpsyg.2015.01198 (2015). 26322003 PMC4533249

[b18] Whitfield-Gabrieli S. & Ford J. M. Default mode network activity and connectivity in psychopathology. Annu Rev Clin Psychol 8, 49–76, doi: 10.1146/annurev-clinpsy-032511-143049 (2012). 22224834

[b19] Canuet L. . Resting-state EEG source localization and functional connectivity in schizophrenia-like psychosis of epilepsy. PLoS One 6, e27863, doi: 10.1371/journal.pone.0027863 (2011). 22125634 PMC3220705

[b20] Razavi N. . Shifted coupling of EEG driving frequencies and fMRI resting state networks in schizophrenia spectrum disorders. PLoS One 8, e76604, doi: 10.1371/journal.pone.0076604 (2013). 24124576 PMC3790692

[b21] Todder D. . The quantitative electroencephalogram and the low-resolution electrical tomographic analysis in posttraumatic stress disorder. Clin EEG Neurosci 43, 48–53 (2012). 22423551 10.1177/1550059411428716

[b22] Bagby R. M., Taylor G. J. & Parker J. D. The Twenty-item Toronto Alexithymia Scale–II. Convergent, discriminant, and concurrent validity. J Psychosom Res 38, 33–40 (1994). 8126688 10.1016/0022-3999(94)90006-x

[b23] Bagby R. M., Parker J. D. & Taylor G. J. The twenty-item Toronto Alexithymia Scale–I. Item selection and cross-validation of the factor structure. J Psychosom Res 38, 23–32 (1994). 8126686 10.1016/0022-3999(94)90005-1

[b24] Derogatis L. The SCL-90-R Manual (Clinical Psychometric Research Unit. Johns Hopkins University School of Medicine, 1977).

[b25] Romei V. . Interhemispheric transfer deficit in alexithymia: a transcranial magnetic stimulation study. Psychother Psychosom 77, 175–181, doi: 10.1159/000119737 (2008). 18332615

[b26] Bressi C. . Cross validation of the factor structure of the 20-item Toronto Alexithymia Scale: an Italian multicenter study. J Psychosom Res 41, 551–559, doi: S0022399996002280 [pii] (1996). 9032718 10.1016/s0022-3999(96)00228-0

[b27] Sarno I., Preti E., Prunas A. & Madeddu F. SCL-90-R: Symptom Checklist 90 R. Versione Italiana Validata e Standardizzata (Giunti O. S. , 2011).

[b28] Dimpfel W., Schober F. & Spuler M. The influence of caffeine on human EEG under resting conditions and during mental loads. Clin Investig 71, 197–207 (1993). 10.1007/BF00180102 8481621

[b29] Kahkonen S., Wilenius J., Nikulin V. V., Ollikainen M. & Ilmoniemi R. J. Alcohol reduces prefrontal cortical excitability in humans: a combined TMS and EEG study. Neuropsychopharmacology 28, 747–754, doi: 10.1038/sj.npp.1300099 (2003). 12655321

[b30] Imperatori C. . Modifications of EEG power spectra in mesial temporal lobe during n-back tasks of increasing difficulty. A sLORETA study. Front Hum Neurosci 7, 109, doi: 10.3389/fnhum.2013.00109 (2013). 23565085 PMC3613724

[b31] Imperatori C. . Aberrant EEG functional connectivity and EEG power spectra in resting state post-traumatic stress disorder: A sLORETA study. Biological Psychology 102C, 10–17, doi: 10.1016/j.biopsycho.2014.07.011 (2014). 25046862

[b32] Pascual-Marqui R. D., Michel C. M. & Lehmann D. Low resolution electromagnetic tomography: a new method for localizing electrical activity in the brain. International Journal of Psychophysiology 18, 49–65, doi: 10.10167-8760(84)90014 (1994). 7876038 10.1016/0167-8760(84)90014-x

[b33] Kreiter A. K. & Singer W. Oscillatory Neuronal Responses in the Visual Cortex of the Awake Macaque Monkey. European Journal of Neuroscience 4, 369–375 (1992). 12106363 10.1111/j.1460-9568.1992.tb00884.x

[b34] Murphy T. H., Blatter L. A., Wier W. G. & Baraban J. M. Spontaneous synchronous synaptic calcium transients in cultured cortical neurons. The Journal of Neuroscience 12, 4834–4845 (1992). 1361198 10.1523/JNEUROSCI.12-12-04834.1992 PMC6575780

[b35] Grave de Peralta-Menendez R. & Gonzalez-Andino S. L. A critical analysis of linear inverse solutions to the neuroelectromagnetic inverse problem. IEEE Transactions on Bio-Medical Engineering 45, 440–448, doi: 10.1109/10.664200 (1998). 9556961

[b36] Grave de Peralta Menendez R., Gonzalez Andino S. L., Morand S., Michel C. M. & Landis T. Imaging the electrical activity of the brain: ELECTRA. Human brain mapping 9, 1–12 (2000). 10643725 10.1002/(SICI)1097-0193(2000)9:1<1::AID-HBM1>3.0.CO;2-# PMC6871828

[b37] Pascual-Marqui R. D. . Assessing interactions in the brain with exact low-resolution electromagnetic tomography. Philosophical Transactions of the Royal Society A: Mathematical, Physical & Engineering Sciences 369, 3768–3784, doi: 10.1098/rsta.2011.0081 (2011). 21893527

[b38] Pagani M. . Neurobiological correlates of EMDR monitoring - an EEG study. PLoS One 7, e45753, doi: 10.1371/journal.pone.0045753 (2012). 23049852 PMC3458957

[b39] Canuet L. . Resting-state network disruption and APOE genotype in Alzheimer’s disease: a lagged functional connectivity study. PLoS One 7, e46289, doi: 10.1371/journal.pone.0046289 (2012). 23050006 PMC3457973

[b40] Stam C. J., Nolte G. & Daffertshofer A. Phase lag index: assessment of functional connectivity from multi channel EEG and MEG with diminished bias from common sources. Human brain mapping 28, 1178–1193, doi: 10.1002/hbm.20346 (2007). 17266107 PMC6871367

[b41] Pascual-Marqui R. D. Coherence and phase synchronization: generalization to pairs of multivariate time series, and removal of zero-lag contributions. *arXiv:0706*.*1776v3 [stat*.*ME] 12 July 2007*. (http://arxiv.org/pdf/0706.1776) (2007).

[b42] Pascual-Marqui R. D., Michel C. M. & Lehmann D. Segmentation of brain electrical activity into microstates: model estimation and validation. IEEE Transactions on Bio-Medical Engineering 42, 658–665, doi: 10.1109/10.391164 (1995). 7622149

[b43] Nichols T. E. & Holmes A. P. Nonparametric permutation tests for functional neuroimaging: a primer with examples. Human brain mapping 15, 1–25, doi: 10.1002/hbm.1058. (2002). 11747097 PMC6871862

[b44] Holmes A. P., Blair R. C., Watson J. D. & Ford I. Nonparametric analysis of statistic images from functional mapping experiments. Journal of Cerebral Blood Flow & Metabolism 16, 7–22, doi: 10.1097/00004647-199601000-00002 (1996). 8530558

[b45] Friston K. J., Frith C. D., Liddle P. F. & Frackowiak R. S. Comparing functional (PET) images: the assessment of significant change. Journal of Cerebral Blood Flow & Metabolism 11, 690–699, doi: 10.1038/jcbfm.1991.122 (1991). 2050758

[b46] Hayes A. F. Introduction to mediation, moderation, and conditional process analysis: a regression based approach (The Guilford Press, 2013).

[b47] Preacher K. J. & Hayes A. F. Asymptotic and resampling strategies for assessing and comparing indirect effects in multiple mediator models. Behavior Research Methods 40, 879–891, doi: 10.3758/BRM.40.3.879 (2008). 18697684

[b48] Deco G., Jirsa V. K. & McIntosh A. R. Emerging concepts for the dynamical organization of resting-state activity in the brain. Nat Rev Neurosci 12, 43–56, doi: 10.1038/nrn2961 (2011). 21170073

[b49] Klimesch W. EEG alpha and theta oscillations reflect cognitive and memory performance: a review and analysis. Brain Res Brain Res Rev 29, 169–195 (1999). 10209231 10.1016/s0165-0173(98)00056-3

[b50] Aftanas L. I. & Golocheikine S. A. Human anterior and frontal midline theta and lower alpha reflect emotionally positive state and internalized attention: high-resolution EEG investigation of meditation. Neurosci Lett 310, 57–60, doi: S0304-3940(01)02094-8 (2001). 11524157 10.1016/s0304-3940(01)02094-8

[b51] Wingbermuhle E., Theunissen H., Verhoeven W. M., Kessels R. P. & Egger J. I. The neurocognition of alexithymia: evidence from neuropsychological and neuroimaging studies. Acta Neuropsychiatr 24, 67–80, doi: 10.1111/j.1601-5215.2011.00613 (2012). 26952949

[b52] Vogt B. A., Finch D. M. & Olson C. R. Functional heterogeneity in cingulate cortex: the anterior executive and posterior evaluative regions. Cereb Cortex 2, 435–443 (1992). 1477524 10.1093/cercor/2.6.435-a

[b53] Leech R., Kamourieh S., Beckmann C. F. & Sharp D. J. Fractionating the default mode network: distinct contributions of the ventral and dorsal posterior cingulate cortex to cognitive control. The Journal of Neuroscience 31, 3217–3224, doi: 10.1523/JNEUROSCI.5626-10.2011 (2011). 21368033 PMC6623935

[b54] Fransson P. & Marrelec G. The precuneus/posterior cingulate cortex plays a pivotal role in the default mode network: Evidence from a partial correlation network analysis. Neuroimage 42, 1178–1184, doi: 10.1016/j.neuroimage.2008.05.059 (2008). 18598773

[b55] Mantani T., Okamoto Y., Shirao N., Okada G. & Yamawaki S. Reduced activation of posterior cingulate cortex during imagery in subjects with high degrees of alexithymia: a functional magnetic resonance imaging study. Biol Psychiatry 57, 982–990, doi: 10.1016/j.biopsych.2005.01.047 (2005). 15860338

[b56] Kano M. . Specific brain processing of facial expressions in people with alexithymia: an H2 15O-PET study. Brain 126, 1474–1484 (2003). 12764066 10.1093/brain/awg131

[b57] Berthoz S. . Effect of impaired recognition and expression of emotions on frontocingulate cortices: an fMRI study of men with alexithymia. Am J Psychiatry 159, 961–967, doi: 10.1176/appi.ajp.159.6.961 (2002). 12042184

[b58] Karlsson H., Naatanen P. & Stenman H. Cortical activation in alexithymia as a response to emotional stimuli. Br J Psychiatry 192, 32–38, doi: 10.1192/bjp.bp.106.034728 (2008). 18174507

[b59] Salzman C. D. & Fusi S. Emotion, cognition, and mental state representation in amygdala and prefrontal cortex. Annu Rev Neurosci 33, 173–202, doi: 10.1146/annurev.neuro.051508.135256 (2010). 20331363 PMC3108339

[b60] Miller L. Is alexithymia a disconnection syndrome? A neuropsychological perspective. Int J Psychiatry Med 16, 199–209 (1986). 3804583 10.2190/dae0-ewpx-r7d6-lfny

[b61] Shipko S. Further reflections on psychosomatic theory. Alexithymia and interhemispheric specialization. Psychother Psychosom 37, 83–86 (1982). 7122790 10.1159/000287557

[b62] Tabibnia G. & Zaidel E. Alexithymia, interhemispheric transfer, and right hemispheric specialization: a critical review. Psychother Psychosom 74, 81–92, doi: 10.1159/000083166 (2005). 15741757

[b63] Larsen J. K., Brand N., Bermond B. & Hijman R. Cognitive and emotional characteristics of alexithymia: a review of neurobiological studies. J Psychosom Res 54, 533–541, doi: S002239990200466X (2003). 12781307 10.1016/s0022-3999(02)00466-x

[b64] Bucci W. Symptoms and symbols: a multiple code theory of somatization. Psychoanalytic Inquiry 17, 151–172 (1997).

[b65] Mastrovito D. Interactions between resting-state and task-evoked brain activity suggest a different approach to fMRI analysis. The Journal of Neuroscience 33, 12912–12914, doi: 10.1523/JNEUROSCI.2580-13.2013 (2013). 23926247 PMC6619727

[b66] Miraglia F., Vecchio F., Bramanti P. & Rossini P. M. Small-worldness characteristics and its gender relation in specific hemispheric networks. Neuroscience 310, 1–11, doi: 10.1016/j.neuroscience.2015.09.028 (2015). 26384963

